# Multi-Cavity Nanorefractive Index Sensor Based on MIM Waveguide

**DOI:** 10.3390/nano14211719

**Published:** 2024-10-28

**Authors:** Weijie Yang, Shubin Yan, Ziheng Xu, Changxin Chen, Jin Wang, Xiaoran Yan, Shuwen Chang, Chong Wang, Taiquan Wu

**Affiliations:** 1 School of Electrical and Control Engineering, North University of China, Taiyuan 030051, China; sz202315077@st.nuc.edu.cn (W.Y.); chenchangxin@nuc.edu.cn (C.C.); sz202215044@st.nuc.edu.cn (J.W.); sz202215006@st.nuc.edu.cn (S.C.); 2 School of Electrical Engineering, Zhejiang University of Water Resources and Electric Power, Hangzhou 310018, China; yanxr@zjweu.edu.cn (X.Y.); wangchong@zjweu.edu.cn (C.W.); wutq@zjweu.edu.cn (T.W.); 3 Zhejiang-Belarus Joint Laboratory of Intelligent Equipment and System for Water Conservancy and Hydropower Safety Monitoring, Hangzhou 310018, China; 4 School of Automation and Electrical Engineering, Zhejiang University of Science and Technology, Hangzhou 310023, China; 1240320114@zust.edu.cn

**Keywords:** surface plasmon polaritons, MIM waveguide, Fano resonance, sensitivity

## Abstract

Within this manuscript, we provide a novel Fano resonance-driven micro-nanosensor. Its primary structural components are a metal-insulator-metal (MIM) waveguide, a shield with three disks, and a T-shaped cavity (STDTC). The finite element approach was used to study the gadget in theory. It is found that the adjustment of the structure and the change of the dimensions are closely related to the sensitivity (S) and the quality factor (FOM). Different model structural parameters affect the Fano resonance, which in turn changes the transmission characteristics of the resonator. Through in-depth experimental analysis and selection of appropriate parameters, the sensor sensitivity finally reaches 3020 nm/RIU and the quality factor reaches 51.89. Furthermore, the installation of this microrefractive index sensor allows for the quick and sensitive measurement of glucose levels. It is a positive contribution to the field of optical devices and micro-nano sensors and meets the demand for efficient detection when applied in medical and environmental scenarios.

## 1. Introduction

SPPs, or surface plasmon polaritons, are coupled fluctuations that spread at the metal-medium interface [1]. It results from the interaction of the collective vibration of free electrons (plasma) with electromagnetic waves (light) [2,3,4]. The process of formation occurs when light waves are irradiated onto a metal surface [5]. The light waves’ electric field causes the free electrons on the metal surface to oscillate collectively, which forms a form of electromagnetic wave that travels along the interface [6,7]. This phenomenon occurs mainly at the interfaces where metals (e.g., gold, silver, etc.) are in contact with media (e.g., air, water, etc.) [8,9]. Metal-insulator-metal (MIM) is a type of combination [10] that has a structure similar to that of a sandwich. In addition, SPPs have unique properties that make them useful for a wide range of applications in a number of areas [11]. One of them can be used to fabricate surface-isolated exciton resonance (SPR) sensors [12,13], which enable the detection of biomolecule concentrations and interactions by monitoring the adsorption of molecules at the interface [14,15]. Fano resonance is a resonance phenomenon due to interference effects [16,17], usually involving the interaction of discrete and continuous states [18]. Its resonance peaks usually appear at specific frequencies, while there is no significant response at other frequencies [19,20]. While conventional resonance transmission spectra are symmetrical Lorentzian lines, the Fano resonance is characterized by an asymmetrical spectrum with sharp changes in phase and amplitude near the resonance point. This characteristic makes the sensor very sensitive to small refractive index changes in the environment, allowing it to detect the presence of trace amounts of biological or chemical substances or changes in concentration. Thus, Fano resonance resonators based on MIM waveguide-coupled structures were created [21], which can achieve ultra-high resolution and fast recognition [22]. Such devices can enhance the light response at specific frequencies by controlling the geometry of the material, the coupling mode, and the environmental parameters, which will greatly enhance the performance of the sensor [23,24,25].

In recent years, this type of sensor has received continuous attention and research [26]. Yiping Sun et al. [27] designed structures with M-shaped and semi-elliptical cavities that can achieve a sensitivity of 1980 nm/RIU and a maximum quality factor of 24.44. Arman Amiri Faghani et al. [28] designed square-crystal nanosheet resonators with S and FOM up to 1140 nm/RIU and 57, respectively. Yunyan Wang et al. [29] designed a semicircular ring sensor with three rectangular cavities with a sensitivity of 2200 nm/RIU and a maximum FOM value of 7.7 × 10^5^. Chang Liu et al. [30] created a multifunctional MIM device. In the end, 1025 nm/RIU of sensitivity and 31.6 quality factor were attained by the device. Most of these work with non-through waveguide coupling; the incident light cannot reach directly and must be coupled to a resonant cavity by way of forming a pathway. When light propagates to these locations, some of the energy is reflected, reducing the efficiency of energy transfer. In our work, a penetrating waveguide is used to maintain the efficient energy transfer, and a more efficient Fano resonance can be realized. In addition, the plasma-waveguide cavity structure has advantages in integration, high performance, and scalability over other forms of Fano resonance sensors. This work studies and designs a novel wavelength sensor. The main MIM waveguide, a shield with three disks, and a T-shaped cavity (STDTC) make up the sensor. In order to be able to obtain an excellent performance index, the structure of the device is analyzed and studied to explore the intrinsic mechanism of action, which provides a theoretical basis for better design of the resonator. Meanwhile, experimental analysis was carried out for cavities of different sizes. By fitting and analyzing the data, it is found that the size of the added cavity is directly related to the transmission characteristics of the resonator. The best performance parameter is obtained under the satisfaction of sensitivity, quality factor, and transmittance. Not only that, the performance of the sensor was experimentally tested for practical applications, and finally a refractive index sensor capable of glucose concentration detection was obtained.

## 2. Materials and Methods

**Figure 1:**
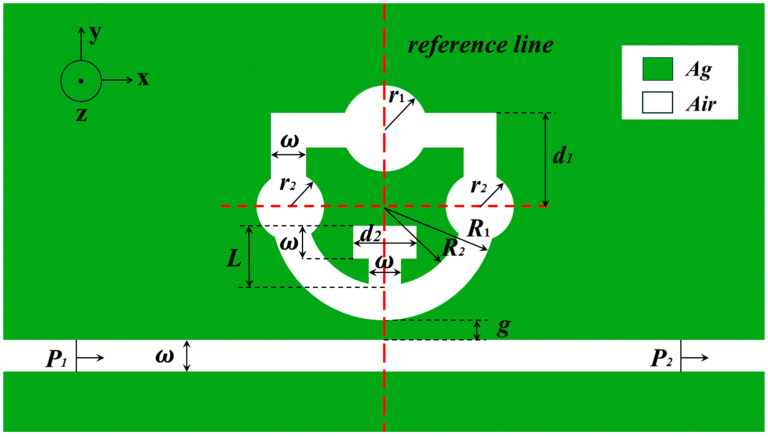
Two-dimensional schematic diagram of a shield-shaped structure with three discs and a T-shaped cavity.

Figure 1 illustrates the 2D structural model designed in this study, which mainly consists of the STDTC structure and the MIM waveguide. The design of the shield ring cavity mainly contains the upper n-shaped structure and the lower semicircular ring, in addition to a large disk, two small disks, and a T-shaped cavity. The overall structure exhibits vertical center symmetry with two reference lines crossing through the center of the semicircular ring. To achieve particular electromagnetic field distribution characteristics and minimize undesirable interference, only transverse magnetic field (TM) modes are stimulated, as indicated by the 50 nm width ω of the ring and MIM waveguide. The remaining parameters of the model in Figure 1 are defined in detail in Table 1.

In this study, a laser is used as a light source, and the wavelength and frequency of the laser can be precisely controlled so that it can interact with the structure of the sensor to improve sensitivity and accuracy. The injection port of the laser is shown in Figure 1, and the light excites the sensor from left to right. During the preparation process, silver was chosen as the medium to promote the excitation and propagation of surface-isolated excitations, which enhances the sensor’s measurement effectiveness. In addition, the low power consumption of silver further enhances its application advantages. Silver nanolayers are formed by depositing a silver film on a silica substrate, which is then etched using a focused ion beam. The Debye–Drew dispersion model below can be used to calculate the dielectric constant of silver [31]:

(1) $$\varepsilon_{Ag}=\varepsilon_{\infty }-\frac{\omega_{p}^{2}}{\omega^{2}+i\gamma \omega }$$

where $\varepsilon_{\infty }$ is the dielectric constant of 3.7 at silver at infinite frequency, $\omega_{p}$ is 1.37 × 10^16^ rad/s, and γ is 0.27 × 10^14^ rad/s.

SPPs excite TM mode during conduction [32], which can be stated using the subsequent formula:

(2) $$\tanh\left(k\omega \right)=\frac{-2kp\alpha_{c}}{\left(k^{2}+p^{2}k^{2}\right)}$$

where p is the ratio of the dielectric constant of the insulator and the metal, α=√k, K=2π⁄λ, *k* is the wave number, and λ is the wavelength.

In addition, the purpose of assessing the benefits of the created model, sensitivity, and quality factors are the key references. Sensitivity is used to measure how well the sensor responds to changes in the measured physical quantity, while the quality factor reflects the combined performance of the sensor’s sensitivity and resolution. The sensitivity and quality factor can be represented using a formula provided as follows [33]:

(3) $$S=\frac{\Delta \lambda }{\Delta n}$$

(4) $$FOM=\frac{S}{FWHM}$$

where *FWHM* is the half-width of the transmission spectral curve, *FOM* is the ratio of the sensitivity to the half-width, and *S* is the ratio of the wavelength at the position of varying inclination to the corresponding varying refractive index.

In order to analyze the constructed model accurately, flexibly, and comprehensively, we used a finite element (FEM) algorithm for the STDTC, which divides the model into a number of finite polygonal cells and performs independent calculations for each cell. We have constructed a 2D model structure of STDTC using COMSOL Multiphysics 5.6 and analyzed it to study the transmission characteristics and performance of nanorefractive index sensors. Firstly, the parameters of the electromagnetic properties of silver as well as other materials were set in the software. Then the parameters, such as wavelength and frequency, when the laser is used as a light source are set to adjust the incident port of light. To ensure the accuracy of the calculation results, ultra-fine triangular meshing was performed on the model and applied mainly to the waveguide and the structure of the design. In addition, a perfect match layer is set up around the designed structure, and boundary conditions are set up in the software, which can absorb the light at the boundary of the region to ensure the accuracy of the simulation results. Most importantly, the geometry and dimensions of each part are accurately set in the software based on the actual physical model. Finally, the parameters and values of the solver, result, data set, image, and other modules in the software are configured. Solving calculations are carried out to obtain the distribution of relevant physical quantities of the model, such as transmission spectra, magnetic and electric field distribution maps, and other results.

## 3. Results and Analysis

**Figure 2:**
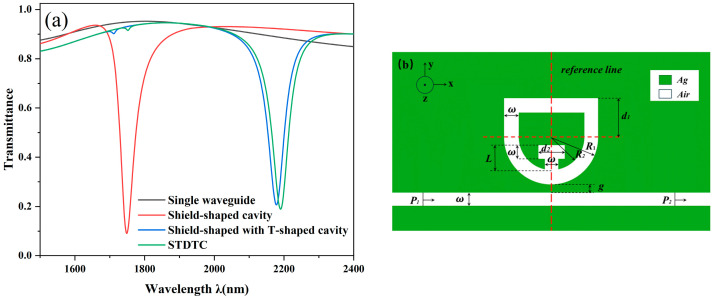
(**a**) Transmission spectra of different structures; (**b**) Shield structure with T-cavity.

To find the optimal performance, it is necessary to examine how several resonant cavities with varying specifications and architectures affect the transmission properties. We compared the different structures and investigated their transmission characteristics. The default parameters of the system are as follows: R_1_ = 210 nm, R_2_ = R_1_ − 50 nm, r_1_ = 60 nm, r_2_ = 50 nm, d_1_ = 190 nm, d_2_ = 180 nm, g = 10 nm, and L = 110 nm. Figure 2a demonstrates the transmission spectra of a single MIM waveguide, a shield-shaped cavity, a shield structure with a T-shaped cavity, and the STDTC; the curves of these different structures are shown in black, red, blue, and green, respectively. Figure 2b, on the other hand, shows the shield structure with a T-shaped cavity only.

Firstly, it can be observed that the single waveguide curve is characterized by a high and almost horizontal transmittance, in which light can propagate relatively easily with low energy loss. The waveguide has a strong response to external excitation signals over a wide frequency range and can transmit light signals over a wide range of wavelengths, so its curve can be considered as a continuous broadband mode. Except for the waveguide curves, the curves of the other structures show a clear inclination, especially at the transmission inclination position, which is characterized by low transmittance. The resonance widths (often expressed as FWHM) of these curves are relatively small and respond very strongly to light only in a specific small frequency range; hence, these curves are called discrete narrowband modes. For example, the resonance profile corresponding to the structure of Figure 2b is asymmetric, which can be simply decomposed into narrowband modes generated by the waveguide and broadband modes generated by the shield structure with a T-shaped cavity. The Fano resonance phenomenon, which eventually leads to asymmetric Fano resonance curves, is caused by the interplay between the broadband modes and the narrowband modes.

Next, we discuss the effect of the introduction of different cavities on the whole structure. The single-shield structure has a wavelength of 1749 nm at the tilt position, when the transmission is extremely low, while the addition of the T-shaped cavity increases the wavelength at the tilt position to 2178 nm, which still exhibits low transmission. The comparison of the two visible curves has a significant red shift, which indicates that the overall structure has been significantly improved in sensitivity, while the FWHM is slightly increased. This means that the light wave can be maintained for a longer period of time near the resonance frequency, allowing the designed sensor to achieve a stronger signal and higher sensitivity.

Subsequently, we introduced the T-shaped cavity along with three disc-shaped cavities at the same time (the STDTC structure), as shown in the green curve, with the wavelength at the tilted position of 2190 nm for the whole system on the original basis. At this point, compared to the shield-shaped structure with only T-shaped cavities, the STDTC not only slightly reduces the transmittance but also enhances the sensitivity of the structure, and the coupling of the light has been improved as well. This suggests that both the disk-shaped cavity and the T-shaped cavity can improve the transmission characteristics, although the contribution of the disk cavity is slightly weaker compared to that of the T-shaped cavity. In the end, by optimizing the original resonator, we have successfully obtained a superior STDTC structure.

**Figure 3:**
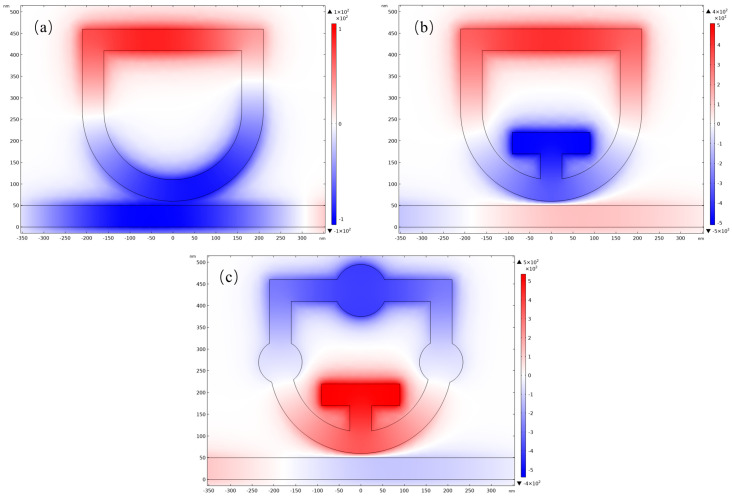
Magnetic field strength distribution (**a**) Single shield cavity; (**b**) Shield with T-cavity; (**c**) STDTC.

In order to better understand the effect of adding a cavity on the whole resonator, the intrinsic mechanism needs to be investigated. As shown in Figure 3, the standardized magnetic field distributions of the resonator are demonstrated. From the figure, it can be seen that each standardized magnetic field distribution has both positive and negative phase states, which indicates the same pattern for different structures. The magnetic field distributions are symmetric around the centerline of the resonator. Taking Figure 3a as an example, the upper phase of the single shield-shaped cavity is in a positive phase while the lower one is in an anti-phase, and this interaction between positive and anti-phase affects the Fano resonance characteristics.

Secondly, comparing Figure 3b and Figure 3c with Figure 3a, respectively, it can be observed that after the addition of the T-shaped cavity, a stronger magnetic field collects inside the cavity than before, which is one of the reasons for the response change. Another factor is that the intensity inside the waveguide starts to decrease after the structure change, indicating that more light enters the STDTC structure. This indicates that more light is involved in the coupling process, and the field strengths of the forward and reverse phases are higher, which enhances the degree of response of the resonance. Meanwhile, comparing Figure 3b with Figure 3c, the disc cavity in the structure of Figure 3c also gathers a higher intensity of light, which further enhances the effect than when only the T-shaped cavity is incorporated. In addition, the strength of the ability to capture surface plasma waves also determines the transmittance and the STDTC structure has a stronger ability to capture SPPs, resulting in a lower transmittance.

Next, we need to adjust the parameters of the system to study its effect on the performance of the resonator. In this way, the performance of the system under this structure can be maximized, and at the same time, it is easy to adjust the parameters to meet individual needs during the actual application. For the resonator, sensitivity and the quality factor are important references.

**Figure 4:**
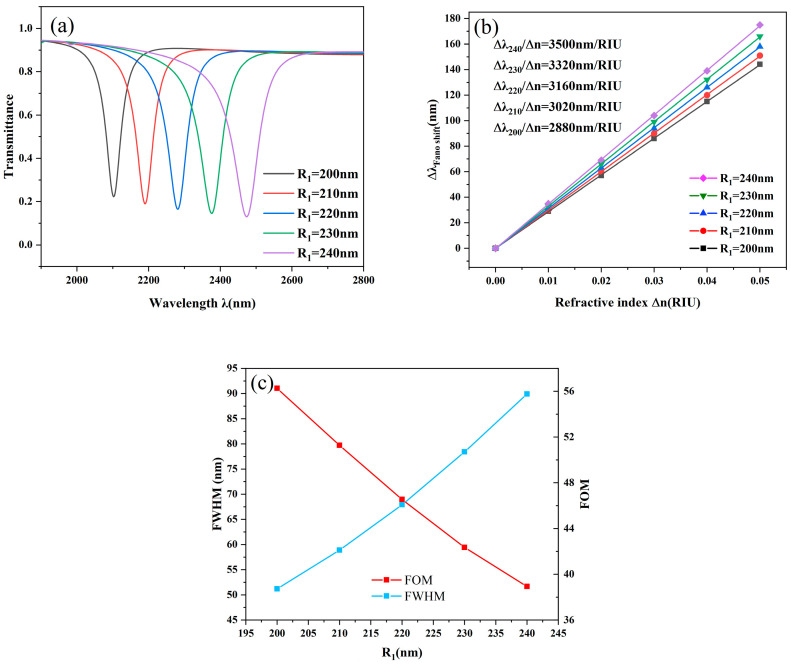
(**a**) Transmission curves with varying R_1_ lengths; (**b**) Sensitivity fit lines with varying R_1_ lengths; (**c**) Comparing FWHM with FOM for various R_1_ lengths.

First, the size of the radius R_1_ of the STDTC was investigated (the difference between R_1_ and R_2_ was kept constant at 50 nm). As can be seen in Figure 4a, as the radius of R_1_ is sequentially increased from 200 nm to 240 nm, the transmission spectrum is clearly red-shifted, with the curve inclination wavelength sequentially increasing from 2100 nm to 2500 nm. At the same time, the transmittance decreases marginally, and the FWHM begins to increase gradually, which is even more clearly observable in Figure 4b. Figure 4b shows the fitting data with respect to the radius size; different sizes of radius correspond to the sensitivity; the radius increases while the sensitivity also increases, and this also confirms the fact that with the red shift of the transmission spectrum, the sensitivity also increases. The red line in Figure 4c shows another performance index, FOM, which is the opposite of sensitivity and shows a decreasing state, so it is necessary to balance the sensitivity and FOM in practical applications. Finally, the optimal performance of the resonator is obtained when R_1_ = 210 nm, and the sensitivity at this time is 3020 nm/RIU, with a quality factor of 51.3.

**Figure 5:**
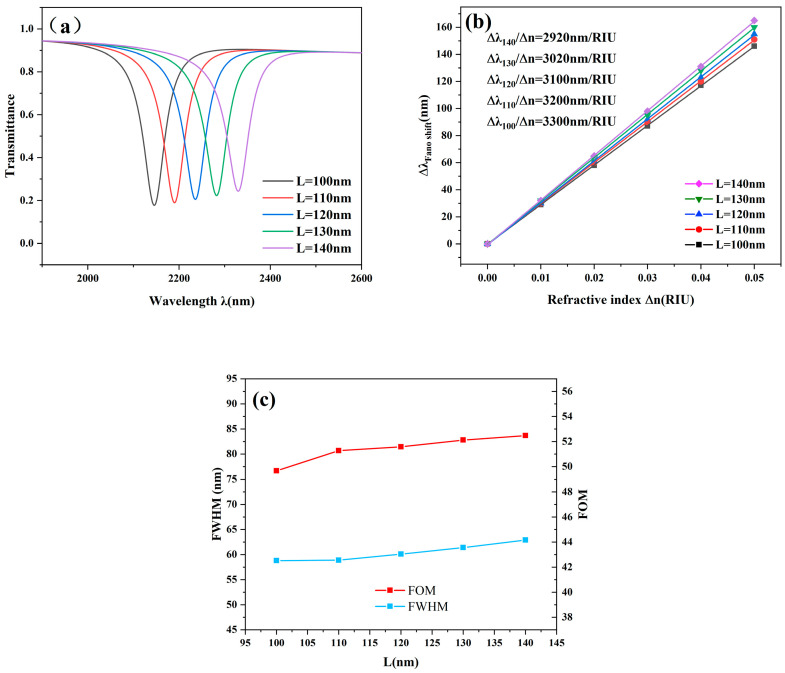
(**a**) Transmission curves with varying L lengths; (**b**) Sensitivity fit lines with varying L lengths; (**c**) Comparing FWHM with FOM for various L lengths.

After that, we examined how the additional cavities’ dimensions affected the overall function. Firstly, the length L of the T-shaped cavity was varied, for which it was set to 100, 110, 120, 130, and 140 nm, respectively. As shown in Figure 5a, the curve redshifts, the dip wavelength shifts from 2100 nm to 2400 nm, the L length grows, and the radius R_1_ modifies. However, its transmittance increases. This indicates that although increasing the T-shaped cavity has a great improvement on the system, excessive increase in size cuts the strength of the Fano resonance. Based on Figure 5b, it can be analyzed that the larger size of L has a good effect on improving the device sensitivity. When L = 140 nm, its sensitivity can reach 3300 nm/RIU, but its transmittance is relatively high at nearly 0.3. FOM and FWHM slightly increase with the length of L but basically remain stable, so when selecting the appropriate length of L, it is mainly to satisfy the requirements of transmittance. So, when L = 110 nm is the most suitable parameter, its corresponding S and FOM are 3020 nm/RIU and 51.3.

**Figure 6:**
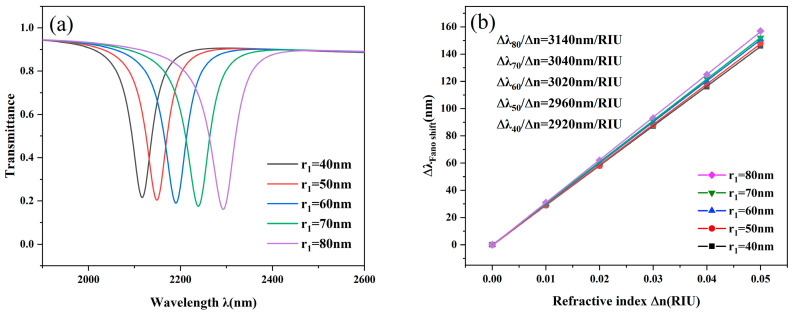
(**a**) Transmission curves with varying r_1_ lengths; (**b**) Sensitivity fit lines with varying r_1_ lengths.

To explore the factors influencing the performance metrics to a greater extent, the variation of the disk cavity r_1_ was then investigated. The r_1_ was increased from 40 nm to 80 nm with an increment of 10 nm. As shown in Figure 6a, the larger the radius of r_1_, the greater its sensitivity is, and its transmittance decreases and vice versa. This means that the larger size of the disk can play a role in reducing the transmittance of the resonator, increasing the intensity of the Fano resonance, and improving the response speed of the sensor. We find that the model is in the most excellent state when the size of r_1_ is chosen to be moderate (60 nm). Besides, the transmission spectra of the disk cavity are the same as those of the T-shaped cavity, and the FWHM and FOM do not change much and basically remain stable.

**Figure 7:**
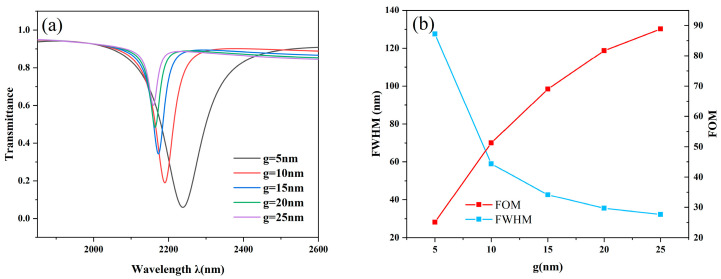
(**a**) Transmission curves with varying g lengths; (**b**) Comparing FWHM with FOM for various g lengths.

Here, we investigate the effect of the parameter g (the distance between the STDTC and MIM waveguides) on the coupling effect, since its magnitude directly determines the coupling effect. We discover that the change of the g parameter exhibits a clear regularity after carefully examining the experimental data. As shown in Figure 7a, the inclination wavelength decreases slightly with the increase of the coupling distance, showing a blue-shifted trend. However, as shown by the blue line in Figure 7b, the FWHM decreases drastically at first and then decreases slowly, while the FOM curve represented by the red line shows the opposite trend. A comprehensive analysis shows that the performance of FWHM and FOM needs to be taken into account when determining the coupling distance. If the distance is too large, it will lead to too long half-width, resulting in a decrease in the quality factor, while if the distance is too small, it will lead to too high inclination transmittance. Therefore, choosing g = 10nm can optimize all kinds of indexes.

**Figure 8:**
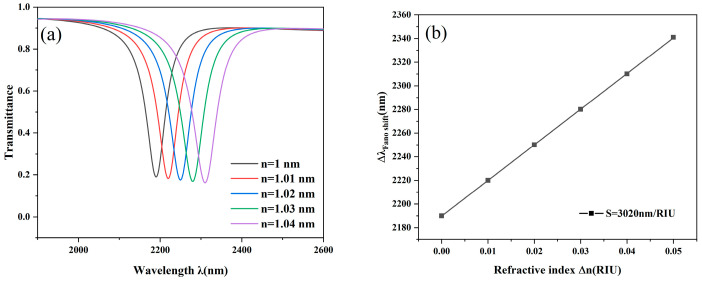
(**a**) Transmission spectra for various indexes of refraction; (**b**) Refractive index sensitivity fit lines.

During the application of the device, the refractive index of the measured substance is not constant, so it is necessary to analyze the performance of the device under different refractive indices. The results are displayed in Figure 8a as we examined the impact of various optical index values on the transmission spectra by gradually setting n from 1 to 1.05. As the refractive index increases, the resonance wavelength shifts significantly, while the transmission decreases slightly. In addition, by fitting the data to the inclined wavelengths with different refractive indices, Figure 8b shows that the change pattern conforms to a linear relationship, and the shifts of the curves exhibit an equidistant redshift. This discovery supports the impact of the refractive index on transmission properties and offers a theoretical foundation for the device’s flexibility in real-world scenarios.

## 4. Applications

The nanoparticle sensitivity of metals and other dielectric hybrid materials can reflect the degree of sensitivity with which the device detects small changes in the relevant factors in the environment [34,35]. When a biological or chemical substance is present in the surrounding environment, it causes changes in the location and intensity of the peaks. By measuring these changes, the presence and concentration of the biological or chemical substance can be detected [36]. In addition, by changing the shape, size, and internal structure, it is possible to optimize the response to environmental changes by adjusting the optical coupling to increase the sensitivity [37,38]. In this paper, we focus on the application of the designed STDTC structured device for the detection of glucose content. The detection of glucose concentration is important in several fields, including medical diagnostics, biochemical research, and the food industry [39]. We fill the air band portion of the resonator with a glucose solution. The incident light enters the glucose solution through the P_1_ port of the MIM waveguide, and the outgoing light is output from the other side. By calculating the effect of different glucose concentrations on the coupling intensity, the detection of glucose concentration can be realized. Refractive indices vary across glucose solution concentrations, and variations in refractive index also result in variations in the Fano resonance effect. As a result, the Fano resonance spectra are very responsive to environmental changes. You can represent the link between refractive index and glucose levels as follows:

(5) n=0.0001189C+1.3323055

where C denotes the glucose solution’s concentration and n stands for the refractive index. Setting the concentration to 0, 40, 80, 120, 160, and 200 g/L in order, it can be calculated that its corresponding refractive index is 1.33231, 1.33706, 1.34181, 1.34657, 1.35133, and 1.35609 in order.

**Figure 9:**
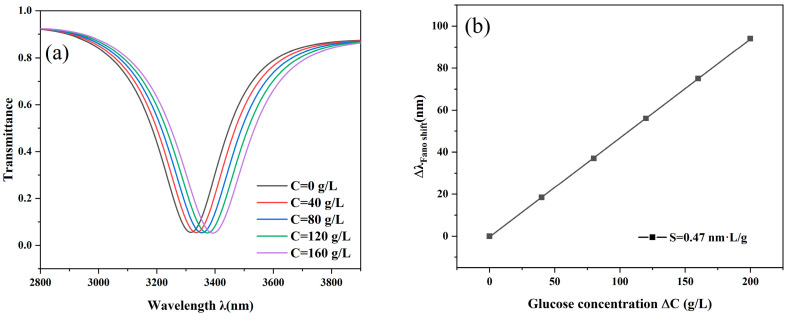
(**a**) Transmission spectrum of glucose concentration; (**b**) Fitted line of glucose concentration.

The transmission spectrum exhibits clear red-shift phenomena and the glucose concentration varies, as illustrated in Figure 9a. This suggests that the sensor is sensitive to the detection of glucose and has the capacity to precisely distinguish between concentrations, which is useful for application. In addition, by analyzing the relationship between the concentration of glucose solution and wavelength, a fitting relationship between the two was obtained. By studying the relationship between the two, the sensitivity (metal/dielectric nanoparticle sensitivity) used for the biosensor can be obtained, which can be expressed as [40]:

(6) $$S=\frac{\Delta \lambda }{\Delta C}$$

This sensor has a predicted sensitivity of 0.47 nm∙L/g for the measurement of glucose concentration, as illustrated in Figure 9b.

Table 2 demonstrates the performance metrics of the structures studied in this paper and compares them with other related structures. It is crucial to remember that different methodologies are used in some literature to compute the quality factor FOM, which could cause significant variations in the outcomes. 

## 5. Conclusions

This work proposes a micro-nano sensor based on Fano resonance, which is made up of a MIM waveguide with three disk T-cavities arranged in a shield-shaped cavity. The structure and size variations of the device are discovered to be closely related to the sensitivity and quality factor after a theoretical analysis using the finite element approach. Comparing the transmission characteristics of different structures, it is found that the interaction of broadband and narrowband modes of the waveguide profile triggers the Fano resonance phenomenon. The addition of a T-shaped cavity and a disk-shaped cavity increases the sensitivity of the system with better optical coupling. Changing the resonator structure affects the resonance frequency, coupling effect, and the ability to capture SPPs, thus optimizing the sensor performance. The effect of system parameters on the performance of the resonator was investigated, and it was concluded that the optimal performance of the resonator was achieved at R_1_ = 210 nm, L = 110 nm, r_1_ = 60 nm, and g = 10 nm, with a sensitivity of 3020 nm/RIU and a quality factor of 51.3. This nanorefractive index sensor can be used to detect the concentration of glucose, which is characterized by its sensitivity in response and its ability to accurately discriminate the concentration.

## Figures and Tables

**Table 1:** Definition of parameters in the structure.

Parameters	Definition
R_1_	Outer radius of a semicircular ring
R_2_	Inner radius of a semicircular ring
r_1_	Radius of the large disk
r_2_	Radius of a small disk
d_1_	Width of the n-shaped ring
d_2_	Length of T-shaped cavity rectangle
L	Width of T-shaped cavity rectangle
g	Coupling distance
ω	Width of waveguide
P_1_	Input port of the light
P_2_	Output port of the light

**Table 2:** Comparison of different structures.

Reference	Sensitivity	FOM	Nanoparticle Sensitivity
[27]	1980 nm/RIU	24.44	30.2 nm∙mL/mg (triglyceride)
[28]	1140 nm/RIU	57	
[29]	2200 nm/RIU	7.7 × 10^5^	0.9 nm/°C (temperature)
[30]	1025 nm/RIU	31.6	
[39]	1085 nm/RIU	7667	0.136 nm∙L/g (glucose)
This work	3020 nm/RIU	51.3	0.47 nm∙L/g (glucose)

## Data Availability

The original contributions presented in the study are included in the article, further inquiries can be directed to the corresponding author.
